# Performance counter dataset for behavioural biometric purpose

**DOI:** 10.1016/j.dib.2023.109999

**Published:** 2023-12-21

**Authors:** Cesar Andrade, Hendrio Bragança, Eduardo Feitosa, Eduardo Souto

**Affiliations:** Institute of Computing, Federal University of Amazonas, Amazonas, Brazil

**Keywords:** Continuous authentication, Behavioural biometrics, Performance counters, Machine learning

## Abstract

In the pursuit of advancing research in continuous user authentication, we introduce COUNT-OS-I and COUNT-OS-II, two distinct performance counter datasets from Windows operating systems, crafted to bolster research in continuous user authentication. Encompassing data from 63 computers and users, the datasets offer rich, real-world insights for developing and evaluating authentication models. COUNT-OS-I spans 26 users in an IT department, capturing 159 attributes across diverse hardware and software environments over 26 h on average per user. COUNT-OS-II, on the other hand, encompasses 37 users with identical system configurations, recording 218 attributes per sample over a 48-hour period. Both datasets utilize pseudonymization to safeguard user identities while maintaining data integrity and statistical accuracy. The well-balanced nature of the data, confirmed by comprehensive statistical analysis, positions these datasets as reliable benchmarks for the continuous user authentication domain. Through their release, we aim to empower the development of robust, real-world applicable authentication models, contributing to enhanced system security and user trust.

Specifications Table

**Table:** 

Subject	*Computer Science.*
Specific subject area	*Data Mining and Machine Learning.*
Data format	Analysed, Filtered.
Type of data	The data consists of tables and lists containing a feature set related to Windows OS Performance Counters, organized, and separated by users.
Data collection	Data was collected from an extensive selection of performance counters accessible on the Windows operating system using the native Performance Monitor tool in the Windows operating system, *Perfmon*[1], and a Python tool was developed to preprocess the data (selection of relevant counters, standardization of counter names) and label the samples. The collections were carried out over 26 h (approximately 4 days of data collection) for Dataset Count-I and 48 h for Dataset Count-II (approximately 8 days of data collection).
Data source location	The data were collected on the computers of the Information Technology Department of a public organization in Brazil.
Data accessibility	Data hosted in public repository [2]. Repository name: **Performance counter for biometrics authentication.** DOI: https://doi.org/10.6084/m9.figshare.24461230.v3 Direct URL to data: https://figshare.com/articles/dataset/Performance_counter_for_biometrics_authentication/24,461,230

## 1 Value of the Data

• Real-World Applicability: The data collected from public organizations in Brazil offers a realistic environment for testing and validating biometric authentication models.
• Comprehensive Data: These datasets encompass various performance counters obtained from the Windows OS, providing a broad perspective on system interactions.
• Variety in System Configurations: COUNT-OS-I includes data from computers with diverse characteristics and configurations, ensuring the models are adaptable to various environments. Conversely, COUNT-OS-II comprises data from computers with similar characteristics and configurations.
• Long-Term Behaviour Analysis: These datasets provide a significant amount of data, averaging 26 h per user for COUNT-OS-I and around 48 h for COUNT-OS-II, enabling the analysis of long-term user behaviour.
• Pseudonymization: Through the application of pseudonymization, user privacy is maintained while preserving the integrity and statistical accuracy of the data.

## 2 Data Description

To advance the field of continuous user authentication, we have meticulously crafted two comprehensive datasets: COUNT-OS-I and COUNT-OS-II, each harboring unique characteristics while sharing common ground in their utility and design principles. These datasets encompass performance counters extracted from the Windows operating system, offering an intricate data set for evaluating and refining authentication models in real-world scenarios.

Both datasets were derived from real-world settings within public organizations in Brazil, e ensuring their relevance and applicability to real-life situations. Volunteers from diverse professional backgrounds participated in the data collection, contributing to the richness and variability of the data. Furthermore, both datasets were collected at a sample rate of every 5 s, providing a dense and detailed view of user interactions and system performance. The commitment to preserving user confidentiality is unwavering across both datasets, with pseudonymization applied meticulously to safeguard individual identities while maintaining data integrity and statistical robustness. The statistical analysis of the number of instances per users in the COUNT-OS-I and COUNT-OS-II datasets can be found in Table 1.

**Table 1:** Statistical analysis of the number of instances by users on COUNT-OS-I, and COUNT-OS-II datasets.

	COUNT-OS-I	COUNT-OS-II
Number of Users	26	37
Mean	18,587.23	34,518.81
Std. Dev.	5188.50	17,709.63
Min.	15,375	4897
25%	16,623	23,157
50%	17,303	33,799
75%	17,536	43,970
Max.	40,523	85,228

## 3 COUNT-OS-I

The COUNT-OS-I dataset was specifically generated in a real-world scenario to evaluate our work on continuous user authentication. This dataset consists of performance counters extracted from the Windows operating system of 26 computers, representing 26 individual users. The data were collected on the computers of the Information Technology Department of a public organization in Brazil.

The participants in this study were volunteers, with aged between 20 and 45 years old, consisting of both males and females. Most of the participants were systems analysts and software developers who performed their routine work activities. There were no specific restrictions imposed on the tasks that the participants were required to perform during the data collection process.

The participants used a variety of software applications as part of their regular work activities. This included web browsers such as Firefox, Chrome, and Edge, developer tools like Eclipse and SQL Developer, office programs such as Microsoft Office Word, Excel, and PowerPoint, as well as chat applications like WhatsApp. It's important to note that the list of applications mentioned is not exhaustive, and participants were not limited to using only these applications.

For the COUNT-OS-I dataset, the data collected is based on computers with different characteristics and configurations in terms of hardware, operating system versions, and installed software. This diversity ensures a representative sample of real-world scenarios and allows for a comprehensive evaluation of the authentication model.

During the data collection process, each sample was recorded at a frequency of every 5 s, capturing system data over a period of approximately 26 h, on average, for each user. This duration provides sufficient data to analyze user behaviour and system performance over an extended period. Each sample in the COUNT-OS-I dataset corresponds to a feature vector comprising 159 attributes. These attributes capture various aspects of system performance, including metrics related to CPU utilization, memory usage, disk activity, network traffic, software APIs and other relevant performance counters. Table 2 presents examples of performance counters that were collected from the different datasets. The complete list of performance counters collected from each dataset can be obtained from the dataset website [2].

**Table 2:** Examples of performance counters attributes used to create the COUNT-OS-I and COUNT-OS-II datasets.

Performance Counter Group Attributes	Type	Description
***PhysicalDisk***		
*Disk Bytes per Second*	Numeric	Measures the rate at which bytes are transferred to or from the disk during read and write operations.
*Disk Write Bytes per Second*	Numeric	Indicates the rate at which bytes are written to the disk per second.
*Disk Read Bytes per Second*	Numeric	Shows the rate at which bytes are read from the disk per second.
*Current Disk Queue Length*	Numeric	Represents the number of disk requests that are currently waiting as well as requests currently being serviced.
*Disk Transfers per Second*	Numeric	The rate at which read and write operations are performed on the disk.
*…*		

***Memory***		
*Percentage of Confirmed Bytes in Use*	Numeric	Shows the percentage of physical memory that is currently in use.
*Page Output per Second*	Numeric	The rate at which pages are written to the disk to free up space in physical memory.
*Page Writes per Second*	Numeric	Indicates the rate at which pages are written to the disk.
*Page Reads per Second*	Numeric	Represents the rate at which pages are read from the disk into physical memory.
*Cache Bytes*	Numeric	Shows the size of the cache memory in bytes.
*…*		

***Processor***		
*DPCs Queued per Second*	Numeric	The number of Deferred Procedure Calls (DPCs) added to the processor's DPC queue per second.
Interrupts per Second	Numeric	Indicates the average number of hardware interrupts that the processor is receiving and servicing in each second.
*Percentage Processor Time*	Numeric	The percentage of time that the processor spends performing non-idle tasks.
*Percentage User Time*	Numeric	Shows the percentage of time that the processor spends executing in user mode (applications).
*Percentage Idle Time*	Numeric	Represents the percentage of time that the processor is idle.
*…*		

***Processor Information***		
*Mean Idle Time*	Numeric	The average time that the processor threads are idle.
*Percentage Privileged Time*	Numeric	Indicates the percentage of time the CPU spends in kernel mode, performing system-level operations.
*C2 Transitions per Second*	Numeric	Number of transitions per second of the processor to the C2 power-saving state.
*C3 Transitions per Second*	Numeric	Number of transitions per second of the processor to the C3 power-saving state.
*C1 Transitions per Second*	Numeric	Number of transitions per second of the processor to the C1 power-saving state.
*…*		

***Network Interface***		
*Bytes Received per Second*	Numeric	The rate at which bytes are received over each network adapter.
*Bytes Sent per Second*	Numeric	Indicates the rate at which bytes are sent over each network adapter.
*%Packets Received Errors*	Numeric	The percentage of received packets that resulted in an error.
*Packets Received Unicast per Second*	Numeric	Number of unicast packets received per second.
*Packets Sent Unicast per Second*	Numeric	Number of unicast packets sent per second.
*…*		
***LogicalDisk***		
*Disk Read Bytes per Second*	Numeric	Rate at which bytes are read from a specific logical disk.
*Disk Write Bytes per Second*	Numeric	Rate at which bytes are written to a specific logical disk.
*Disk Writes per Second*	Numeric	Indicates the rate of write operations on a specific logical disk.
*Disk Reads per Second*	Numeric	Represents the rate of read operations on a specific logical disk.
*%Current Disk Queue Length*	Numeric	Percentage of the current disk queue length relative to the maximum value observed.
*Disk Transfers per Second*	Numeric	Rate at which read and write operations are performed on a specific logical disk.
*…*		

***Process***		
*Page File Bytes*	Numeric	The current size of the page file used by the process.
*IO Data Bytes per Second*	Numeric	Rate at which the process is reading and writing bytes in input-output operations.
*IO Read Operations per Second*	Numeric	Number of input-output read operations performed by the process per second.
*%Thread Count*	Numeric	Percentage of thread count relative to the maximum value observed.
*Percentage Processor Time*	Numeric	Represents the percentage of time that the process's threads spent executing code in user or kernel mode.
*…*		

***Synchronization***		
*Spinlock Acquisitions per Second*	Numeric	The rate at which spin locks are being acquired by the system.
*Spinlock Contentions per Second*	Numeric	Indicates the rate at which threads had to wait to acquire a spin lock.
*IPI Send Software Interrupts per Second*	Numeric	Number of software interrupts sent per second using Inter-Processor Interrupts (IPIs).
*IPI Send Routine Requests per Second*	Numeric	Number of routine requests sent per second using Inter-Processor Interrupts (IPIs).
*Spinlock Rotations per Second*	Numeric	The rate at which threads are acquiring the spin lock after the first attempt.
*…*		

***Users***		
*User*	String	‘u01’-‘u26’ for COUNT-OS-I ‘u01’-‘u37’ for COUNT-OS-II

We apply pseudonymization to hide users' sensitive information by replacing private identifiers with pseudonyms, ensuring the confidentiality of individuals' identities. This technique preserves statistical accuracy and data integrity.

## 4 COUNT-OS-II

This dataset comprises performance counters extracted from the Windows operating system installed on 37 computers. These computers possess identical hardware configurations (CPU, memory, network, disk), operating systems, and software installations. The data collection was conducted within various departments of a public organization in Brazil.

The participants in this study (37 users) were voluntary administration assistants who performed various administrative tasks as part of their routine work activities. No restrictions were imposed on the specific tasks they were assigned. The participants commonly utilized programs such as the Chrome browser and office applications like Office Word, Excel, and PowerPoint, in addition to the WhatsApp chat application.

The data were collected over six days (approximately 48 h), with sample collected at a 5-second interval. Each sample corresponds to a feature vector composed of 218 attributes. In this dataset, we also apply pseudonymization to hide users' sensitive information.

## 5 Data Extraction

**Fig. 1:**
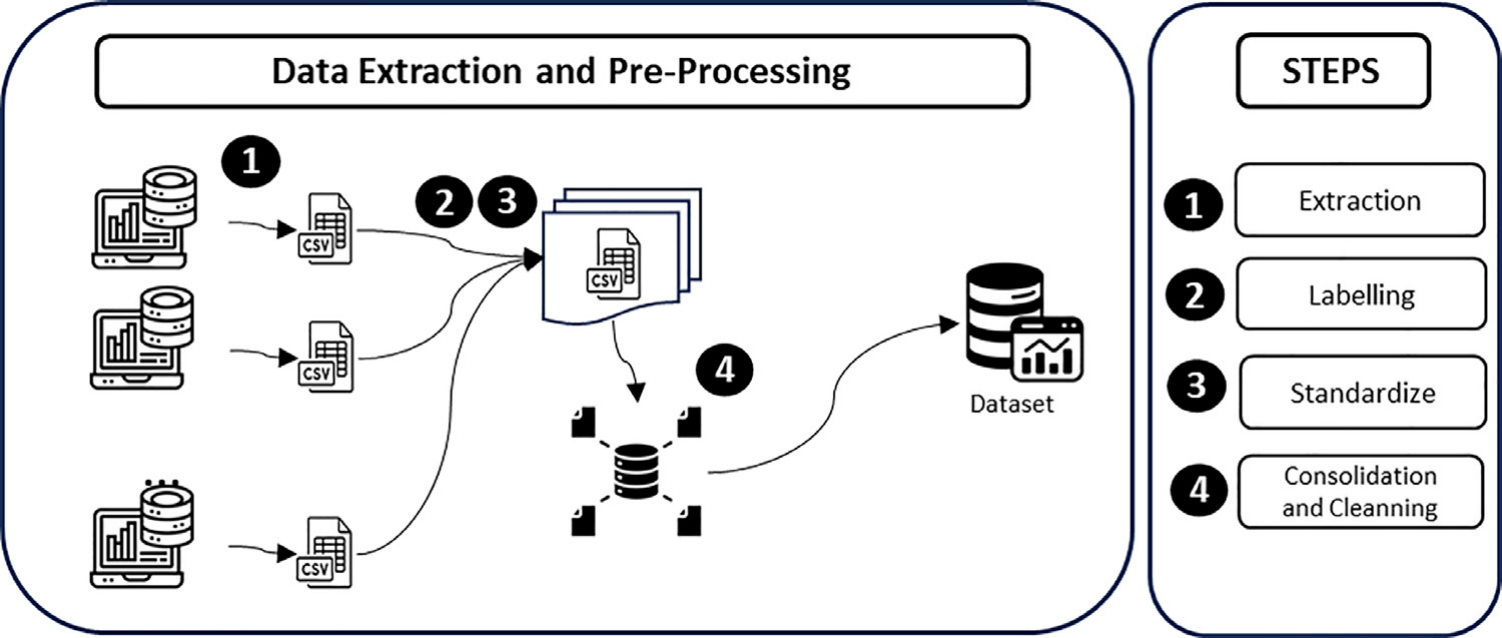
Detailed data extracting and pre-processing steps applied to create the COUNT-OS-I and COUNT-OS-II datasets.

To obtain the Performance Counters features presents in the COUNT-OS-I and COUNT-OS-II datasets, according to Fig. 1, we follow the simple five-step process of turning basic computer performance information into a cleaned and valuable set of data:

1. Data Extraction: In the first step, we utilize *Perfmon* [1], a native performance monitoring tool of the Windows operating system, to collect various system performance metrics. The data is gathered separately for each user, capturing intricate details about their system's performance. Each user's data is then saved into its own CSV file, creating a structured and accessible format for future analysis.
2. Anonymized Labeling: We added an anonymized label to each user within the dataset. This label serves as a unique identifier, ensuring user privacy while allowing us to track and analyze the data across different users.
3. Feature Standardization and Aggregation: In this phase, we remove any special characters that may cause discrepancies in automatic analysis of the data. Additionally, we aggregate performance values by device type. For instance, metrics like 'Network adapter 1 output rate' and 'Network adapter 2 output rate' are combined into a single feature named 'Network adapter output rate - Total,' representing the total network output rate across all network adapters.
4. Data Consolidation and Cleaning: we combine the individual CSV files of all users into a single comprehensive file. During the consolidation process, we began by removing uncommon features among users. This involved excluding attributes not shared by all users to maintain dataset uniformity and prevent user identification through unique attributes. Additionally, we removed features that exhibited no variance in values across users, as these attributes do not significantly contribute to the analysis and could impact the results. As a result of this process, we achieved a cleaned and consistent dataset, containing only the most relevant and diverse features.

## Limitations

Limited Behavioral Context: The datasets focus on performance counters without detailed contextual information about the tasks being performed, which could provide additional insights into user behavior for authentication purposes.

## Ethics Statement

The original authors of the source datasets extracted data samples from performance counters on different computers using the Windows operating system, which were generated by various human subjects. No personally identifiable information was collected as part of the data collection process. Participants provided informed consent for the publication of data and research, and the published data is anonymized. The data collection, conducted following the ethical guidelines established by the Amazonas State Department of Finance, was officially authorized with protocol number 0036–2019/GTEC. The authors declare that they have followed the general ethics rules of scientific research.

## CRediT authorship contribution statement

**Cesar Andrade:** Conceptualization, Methodology, Software, Investigation, Data curation, Writing – review & editing. **Hendrio Bragança:** Conceptualization, Methodology, Investigation, Data curation, Formal analysis. **Eduardo Feitosa:** Conceptualization, Supervision, Writing – review & editing. **Eduardo Souto:** Conceptualization, Supervision, Writing – review & editing.

## Data Availability

Performance counter for biometrics authentication (Original data) (Figshare.com)
